# Notes on Dermatology

**Published:** 1894-11

**Authors:** Isadore Dyer

**Affiliations:** New Orleans, La., Professor of Dermatology in the New Orleans Polyclinic; Lecturer and Clinical Instructor in Skin Diseases, Medical Department Tulane University, Etc.


### NOTES ON DERMATOLOGY.

BY ISADORE DYER, M. D., NEW ORLEANS, LA.,
Professor of Dermatology in the New Orleans Polyclinic; Lecturer and Clinical Instructor in Skin Diseases, Medical Department Tulane University, etc.

THE THIRD INTERNATIONAL CONGRESS OF DERMATOLOGY is to be held in London, July 31st, to August 4th, 1895. Dr. Geo. T. Jackson, 14 East 34th street, New York City, has been appointed Foreign Secretary for the United States.

---

DR. A. D. ROCKWELL, in the *Medical Record* of October 17th, claims to have obtained marked effect with electricity, applied with both the galvanic and faradic currents, in a case of unilateral hyperidrosis. He comments upon this instance of the effect in influencing directly the function of the sympathetic.

---

IN THE OCTOBER NUMBER of the *Journal of Cutaneous and Genito-Urinary Diseases*, Dr. Geo. T. Jackson reports five cases of skin disease treated with thyroid extract. He suggests that the dried gland is the most convenient form for administration, and is best given in capsule. So far from favorable results obtained, there was lack of improvement in the skin, and disagreeable symptoms, more or less severe, from the administration. Dr. Jackson concludes his paper with an analysis of the cases hitherto reported (while, however, he does not include, those csses reported in the French journals, in one instance numbering twenty cases) in all some thirty cases, in which it would appear the conclusion had been reached, excluding the cases of psoriasis reported. This scarcely bears out the statements made by Benjamin Squire a few months ago, or those so enthusiastically ventured by the French dermatologists, who have really done the most and best of the experimenting. Of six cases of my own, only one gave any untoward evidence of the medication. In this case, a psoriatic, there were marked nervous symptoms, anorexia, restlessness and insomnia. All of these disappeared when the extract was discontinued. Two of these six cases were undoubtedly benefited, while two were cured. The other two in no way responded to the medication. One must agree with Dr. Jackson, however, in his comment upon the results obtained in cases of psoriasis. As so many cases of psoriasis get well with little or no treatment, the report of a cure under the thyroid extract, can be of very little value, and especially so, when external treatment has been used in conjunction.

